# *Rickettsia* induces strong cytoplasmic incompatibility in a predatory insect

**DOI:** 10.1098/rspb.2024.0680

**Published:** 2024-07-31

**Authors:** Yuta Owashi, Hiroshi Arai, Tetsuya Adachi-Hagimori, Daisuke Kageyama

**Affiliations:** ^1^ Institute of Agrobiological Sciences, National Agriculture and Food Research Organization (NARO), 1-2 Owashi, Tsukuba, Ibaraki 305-0851, Japan; ^2^ Laboratory of Applied Entomology, University of Miyazaki, 1-1 Gakuenkibanadai-Nishi, Miyazaki 889-2192, Japan

**Keywords:** *Rickettsia*, cytoplasmic incompatibility, reproductive manipulation, endosymbiont, mirid bug

## Abstract

*Rickettsia*, a group of intracellular bacteria found in eukaryotes, exhibits diverse lifestyles, with some acting as vertebrate pathogens transmitted by arthropod vectors and others serving as maternally transmitted arthropod endosymbionts, some of which manipulate host reproduction for their own benefit. Two phenotypes, namely male-killing and parthenogenesis induction are known as *Rickettsia*-induced host reproductive manipulations, but it remains unknown whether *Rickettsia* can induce other types of host manipulation. In this study, we discovered that *Rickettsia* induced strong cytoplasmic incompatibility (CI), in which uninfected females produce no offspring when mated with infected males, in the predatory insect *Nesidiocoris tenuis* (Hemiptera: Miridae). Molecular phylogenetic analysis revealed that the *Rickettsia* strain was related to *Rickettsia bellii*, a common insect endosymbiont. Notably, this strain carried plasmid-encoded homologues of the CI-inducing factors (namely *cifA*-like and *cifB*-like genes), typically found in *Wolbachia*, which are well-known CI-inducing endosymbionts. Protein domain prediction revealed that the *cifB*-like gene encodes PD-(D/E)XK nuclease and deubiquitinase domains, which are responsible for *Wolbachia*-induced CI, as well as ovarian tumour-like (OTU-like) cysteine protease and ankyrin repeat domains. These findings suggest that *Rickettsia* and *Wolbachia* endosymbionts share underlying mechanisms of CI and that CI-inducing ability was acquired by microbes through horizontal plasmid transfer.

## 1. Introduction

*Rickettsia* (Pseudomonadota: Alphaproteobacteria) comprises a group of obligate intracellular bacteria found in eukaryotes. Some of them act as vertebrate pathogens transmitted by arthropod vectors such as mites and fleas, whereas others serve as maternally transmitted arthropod symbionts [1]. Some *Rickettsia* symbionts induce male-killing (MK) in ladybird beetles and leaf-mining beetles, leading to the death of zygotes that would otherwise develop as males [2–4]. Additionally, other *Rickettsia* strains are known to cause parthenogenesis induction (PI) in haplodiploid parasitic wasps, where unfertilized haploid eggs, typically developing as males, undergo diploidization and develop into females [5,6]. These reproductive manipulations are considered advantageous for *Rickettsia* as well as other maternally transmitted symbionts. In addition to MK and PI phenotypes, some invertebrate symbionts, notably *Wolbachia*, induce other types of reproductive manipulations, such as cytoplasmic incompatibility (CI), in which uninfected females produce few or no offspring when they mate with infected males, and feminization (Fem), in which genetic males are phenotypically and functionally feminized. However, it remains unknown whether *Rickettsia* can induce CI or Fem in insect hosts [7,8].

CI is the most common phenotype among symbiont-induced host reproductive manipulations and it leads to the rapid spread of symbionts throughout the host population by severely reducing the number of uninfected offspring [9]. CI is known to be induced by symbionts belonging to five different bacterial genera: *Wolbachia* (Pseudomonadota: Alphaproteobacteria) [7], *Mesenet* (Pseudomonadota: Alphaproteobacteria) [10,11], *Rickettsiella* (Pseudomonadota: Gammaproteobacteria) [12], *Cardinium* (Bacteroidota: Sphingobacteriia) [13] and *Spiroplasma* (Mycoplasmatota: Mollicutes) [14]. As causative factors of CI, *cifA* and *cifB* genes from the *Wolbachia* genome were identified in *Drosophila* and *Culex* species [15,16]. In many cases, *cifA* and *cifB* are found near each other in the prophage region of the *Wolbachia* genome [15–17] and *cifB* encodes PD-(D/E)XK nuclease and/or deubiquitinase as a putative toxic domain. Although the underlying mechanisms remain unclear, either *cifB* alone or both *cifA* and *cifB* modify or poison the sperm of infected males and *cifA* expression in females is sufficient to rescue them [18,19]. Although *cif* homologues have been found in more than 50 strains of *Wolbachia* as well as in some *Rickettsia* and *Mesenet* strains, the CI phenotype has not been observed in *Rickettsia* species [20]. Although it has not been known whether *Rickettsiella* possess *cif* genes, the absence of *cif* homologues in the genomes of CI-inducing *Cardinium* and *Spiroplasma* indicates independent evolution of CI in these symbionts [14,21].

The small green mirid, *Nesidiocoris tenuis* (Hemiptera: Miridae), is a worldwide species commonly used as a biological control agent for agricultural pests [22,23]. Its zoophytophagous trait, which allows it to survive by feeding not only on arthropods but also on plants, can augment its biological control activities but can also cause damage to crops [24,25]. Although it has been reported to retain some symbionts such as *Rickettsia*, *Wolbachia* and *Spiroplasma* [26,27], the phenotype of these symbionts is still unknown.

In this study, we discovered that *Rickettsia* induces strong CI in *N. tenuis*. Genome sequencing revealed that the isolated *Rickettsia* strain was closely related to *Rickettsia bellii* and its genome consisted of a main chromosome and three plasmids, two of which encode homologues of *Wolbachia*-encoded CI effector genes (*cifA*-like and *cifB*-like genes). These findings suggest that *Rickettsia* and *Wolbachia* endosymbionts share underlying mechanisms of CI and that the acquisition of CI capabilities was mediated by horizontal plasmid transfer.

## 2. Material and methods

### (a) Breeding of *Nesidiocoris tenuis*

The isofemale line OW1 infected with *Rickettsia* was established from a female collected in Ibaraki, Japan in 2021 (electronic supplementary material, table S1). In the laboratory, insects were maintained by supplying *Ephestia kuehniella* (Lepidoptera: Pyralidae) eggs (purchased in a frozen state from Agrisect, Ibaraki, Japan) attached to adhesive sheets as the food source and the jade plant, *Crassula ovata* (Saxifragales: Crassulaceae), as the oviposition substrate. A 1 ml glass tube filled with distilled water, which was moderately packed with cotton to prevent water leakage, served as the water source. All breeding experiments were performed under laboratory conditions of 25 ± 1°C, 50 ± 10% humidity and a 14 : 10 h light:dark cycle.

### (b) Antibiotic treatment

To eliminate bacterial symbionts, newly hatched nymphs of the OW1 line were supplied a 1 ml glass tube of distilled water containing 0.05% (w/v) tetracycline hydrochloride together with *E. kuehniella* eggs. The glass tube was moderately packed with cotton to prevent water leakage. During antibiotic treatment, *C. ovata* was not placed in the breeding container except during the oviposition period, to prevent insects from obtaining water from the plant. After four generations of antibiotic treatment, the endosymbiotic bacteria-eliminated OW1 line (OW1^tet^) was used for crossing experiments.

### (c) Crossing experiments

Females and males of the OW1 (*Rickettsia*-infected) and OW1^tet^ (cured) lines were crossed in all possible combinations to compare the number of eggs laid and the hatch rates. Males and females were collected separately from newly emerged adults. A 3–6 day old female was allowed to mate with a male for 7 days and then transferred to a container with a leaf of *C. ovata* for oviposition. Leaves were replaced every 2 days, and oviposition continued for 6 days. Ten days after the last day of oviposition, hatched nymphs were counted and leaves were dissected to count unhatched eggs. The food source was renewed every 2 days during the crossing experiments.

The total number of laid eggs was analysed using the Kruskal–Wallis test. Egg hatch rates were analysed using a generalized linear mixed model (GLMM) with binomial error and a logit-link function. Each mating pair was assigned a random effect. Based on the results of the GLMM, ANOVA was performed for each treatment to estimate the *p*-value using the chi-squared test with Bonferroni correction. Mating pairs with no oviposition were excluded from the analysis of egg hatch rates. The analyses were performed using R v. 4.2.2 [28].

### (d) DNA extraction and PCR

To extract egg DNA, each egg was rinsed with distilled water and squashed in 10 µl of 5% (w/v) Chelex® 100 Resin (Bio-Rad Laboratories, Hercules, CA, USA). Then, 1.0 µl of proteinase K (20 mg ml^−1^) was added to each sample, which was then incubated at 56°C for 2 h followed by 99°C for 3 min. To extract adult DNA, a whole adult body was subjected to DNA extraction using the DNeasy® Blood & Tissue Kit (QIAGEN, Hilden, NRW, Germany) following the manufacturer's protocol, with final elution with 50 μl of Buffer AE (10 mM Tris-Cl, 0.5 mM EDTA; pH 9.0). To detect *Rickettsia*, PCR was performed using KOD FX Neo (TOYOBO, Osaka, Japan) with the *Rickettsia*-specific primers 528-F and 1044-R targeting 16S rRNA [29]. To ensure that DNA was properly extracted, DNA samples were subjected to PCR using LCO1490 and HCO2198 [30], which are universal primers for the mitochondrial cytochrome *c* oxidase subunit I (COI) gene. The PCR protocol consisted of initial denaturation at 94°C for 2 min, followed by 35 cycles of 98°C for 10 s, 60°C (528-F/1044-R) or 50°C (LCO1490/HCO2198) for 30 s and 68°C for 30 s, and a final extension step at 68°C for 7 min.

### (e) Microbiome analysis based on amplicon sequencing

The hypervariable V3–V4 region of the 16S rRNA gene from the founder female of the OW1 line and her three descendants, the subsequent generation of which was used for crossing experiments, were amplified using KOD FX Neo with the primers 1st_PCR_V3V4f_MIX (5′-ACACTCTTTCCCTACACGACGCTCTTCCGATCT-NNNNN-CCTACGGGNGGCWGCAG-3) and 1st_PCR_V3V4r_MIX (5′-GTGACTGGAGTTCAGACGTGTGCTCTTCCGATCT-NNNNN-GACTACHVGGGTATCTAATCC-3′) (where 'NNNNN' is a random sequence of 0–5 bases) according to the protocol of the Bioengineering Lab. Co. (Kanagawa, Japan). The reactions were initiated by denaturation at 94°C for 2 min, followed by 30 cycles of 98°C for 10 s, 55°C for 30 s and 68°C for 30 s, and a final extension step of 68°C for 7 min. After purifying the PCR products using AMPure XP beads (Beckman Coulter, Brea, CA, USA), a second PCR was performed for 12 cycles using the primers 2ndF (5′-AATGATACGGCGACCACCGAGATCTACAC–Index2–ACACTCTTTCCCTACACGACGC-3′) and 2ndR (5′-CAAGCAGAAGACGGCATACGAGAT–Index1–GTGACTGGAGTTCAGACGTGTG-3′). The barcoded amplicons were sequenced on the MiSeq platform (Illumina, San Diego, CA, USA) using the MiSeq Reagent Kit v. 3 (Illumina) to produce 300 bp paired-end reads.

The Illumina reads were extracted using ‘fastx_barcode_splitter’ and trimmed using ‘fastx_trimer’ in the FASTX Toolkit (v. 0.0.14) [31]. Paired-end reads were merged using FLASH (v. 1.2.11) [32]. Denoising and clustering were performed to obtain representative sequences and the feature table using the ‘qiime dada2 denoise-paired’ command in QIIME2 (v. 2022.8) [33]. Taxonomic assignment to the representative sequences was then performed using the ‘qiime feature-classifier classify-blast’ command.

### (f) Genome analysis of the *r*Nten-OW1 strain

DNA from the founder female of the OW1 line was used for library construction using the MGIEasy FS DNA Library Prep Set (MGI Tech Co., Shenzhen, China), MGIEasy Circularization Kit (MGI Tech Co.) and DNBSEQ-G400RS High-throughput Sequencing Kit (MGI Tech Co.) according to the manufacturer's protocol. The constructed library (200 bp paired-end) was sequenced using DNBSEQ-G400 (MGI Tech Co.). Sequenced data, which were mapped to the reference closed *Rickettsia* genome from *N. tenuis* (*r*Nten, AP029035–AP029038) [34] using minimap2 v. 2.17 [35], were converted to the consensus genome using SAMtools v. 1.9 [36]. The genome structures of the resequenced *r*Nten-OW1 strain were visualized using GView [37].

*Wolbachia* Cif proteins [15,16,20] were used as queries to identify Cif homologues in the *r*Nten-OW1 genome via local BLASTp searches (default parameters). The coding sequences of the *cif* genes were further verified manually using Integrative Genomics Viewer (IGV) [38] and Sanger sequencing. The plasmids were annotated using DFAST [39]. The motifs and domains in CifA and CifB homologues encoded by *r*Nten-OW1 were predicted using the HHpred webserver [40] with SCOPe70 (v. 2.08), Pfam-A (v. 35), SMART (v. 6.0) and COG/KOG (v. 1.0), as described by Martinez *et al*. [20].

### (g) Phylogenetic analysis

The 16S rRNA sequences (1494 bp) obtained through next-generation sequencing of the *Rickettsia r*Nten-OW1 isolate were used for phylogenetic analyses. Phylogenetic trees based on the nucleotide sequences were constructed by the maximum-likelihood method using MEGA11 [41]. Kimura's two-parameter model, evaluated using the best fit method, was applied for the calculation.

To infer the phylogeny of the *cif* genes, we obtained the nucleotide sequences of previously identified *cif* homologues [20,42,43]. After manual reannotation as described by Martinez *et al*. [20], *cifA* and *cifB* nucleotide sequences were aligned according to their amino acid translations using MUSCLE implemented in MEGA11 [41]. The aligned data were cleaned using the GBlocks tool in NGPhylogeny.fr [44] to remove weakly conserved regions. Phylogenetic trees based on the cleaned nucleotide sequences were reconstructed by the maximum-likelihood method using MEGA11 [41]. A general time-reversible model, evaluated by the best fit method, was applied for the calculation.

## 3. Results

### (a) *Rickettsia* in *Nesidiocoris tenuis* OW1 line

**Figure 1.**
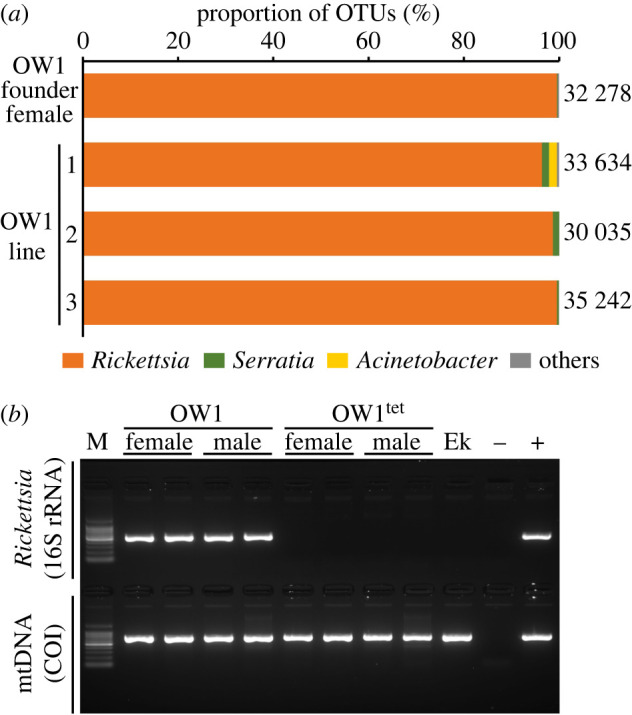
Predominance of *Rickettsia* infection in the OW1 line. (*a*) Proportion of bacterial sequences based on amplicon sequencing of the hypervariable V3–V4 region of the 16S rRNA in the founder female of the OW1 line and three of her descendants. The total number of sequencing reads is presented to the right of each bar and the assigned bacterial taxa are colour-coded at the bottom. (*b*) Examples of diagnostic PCR for *Rickettsia* in the OW1 and OW1^tet^ lines. The PCR amplicons (517 bp) with *Rickettsia*-specific 16S rRNA primers (528-F and 1044-R) are shown at the top and the PCR amplicons (709 bp) with universal mitochondrial cytochrome *c* oxidase subunit I (COI) primers (LCO1490 and HCO2198) are shown below. Ek, *Ephestia kuehniella* eggs, which composed the diet for *Nesidiocoris tenuis* in the laboratory; −, distilled water; +, the founder female of the OW1 line; M, 100 bp DNA ladder (TOYOBO Co., Osaka, Japan).

Amplicon sequencing of the 16S rRNA gene revealed that *Rickettsia* was represented in most reads (99.4%) from the founder female of the OW1 line, which was also observed for her three offspring (96.3–99.5%; figure 1*a*; electronic supplementary material, table S2). *Rickettsia* was also detected in eggs from the OW1 line (24/24), indicating that they were maternally transmitted (electronic supplementary material, table S3). Other minor reads belonged to *Serratia* (0.2–1.5%) and *Acinetobacter* (0–1.5%), which are known gastrointestinal or environmental bacteria [45,46]. *Rickettsia*-specific PCR confirmed that all adults of the OW1 line were *Rickettsia*-positive (24/24) and that all adults of the antibiotic-treated line OW1^tet^ were *Rickettsia*-negative (0/24). *Ephestia kuehniella* eggs, which were used as a food source for *N. tenuis* in the laboratory, were *Rickettsia*-free (figure 1*b*; electronic supplementary material, table S3).

### (b) *Rickettsia* induces cytoplasmic incompatibility in *Nesidiocoris tenuis*

**Figure 2.**
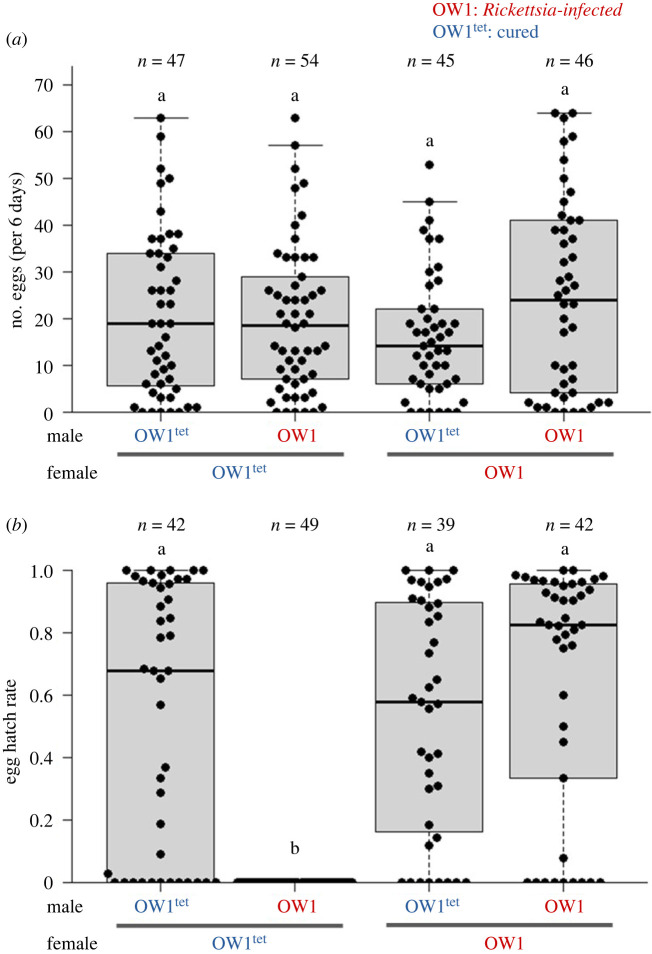
Number of eggs laid and their hatch rate for each mating combination of the OW1 (*Rickettsia*-infected) and OW1^tet^ (cured) lines. (*a*) Each point represents the number of eggs laid by a mating pair over 6 days. The same letter above each combination indicates no significant difference according to the Kruskal–Wallis test (*p* = 0.359). (*b*) Each point represents the hatch rate of eggs produced by a mating pair. Data were analysed using a generalized linear mixed model with binomial error and a logit-link function by assigning each mating pair a random effect. Different letters above each combination indicate significant difference after Bonferroni correction (*p* < 10^−11^). Note that each point in (*b*) corresponds to a point in (*a*).

No significant differences in the numbers of eggs laid were detected for any of the four mating combinations between the OW1 (*Rickettsia*-infected) and OW1^tet^ (cured) lines (Kruskal–Wallis test, *p* = 0.359; figure 2*a*; electronic supplementary material, table S4). Conversely, the egg hatch rates in the offspring produced by crosses between OW1 males and OW1^tet^ females (incompatible combination) were consistently 0% (1072 eggs from 49 crosses) and significantly lower (*p* < 10^−11^) than those of other compatible combinations (median: 53.3–67.8%; figure 2*b*; electronic supplementary material, table S4). In the compatible combinations, mating pairs with 0% egg hatch rates tended to be derived from few eggs (range: 1–13, median: 4; electronic supplementary material, table S4).

### (c) *cif*-like genes are located in plasmids of the cytoplasmic incompatibility-inducing *Rickettsia r*Nten-OW1 line

**Figure 3.**
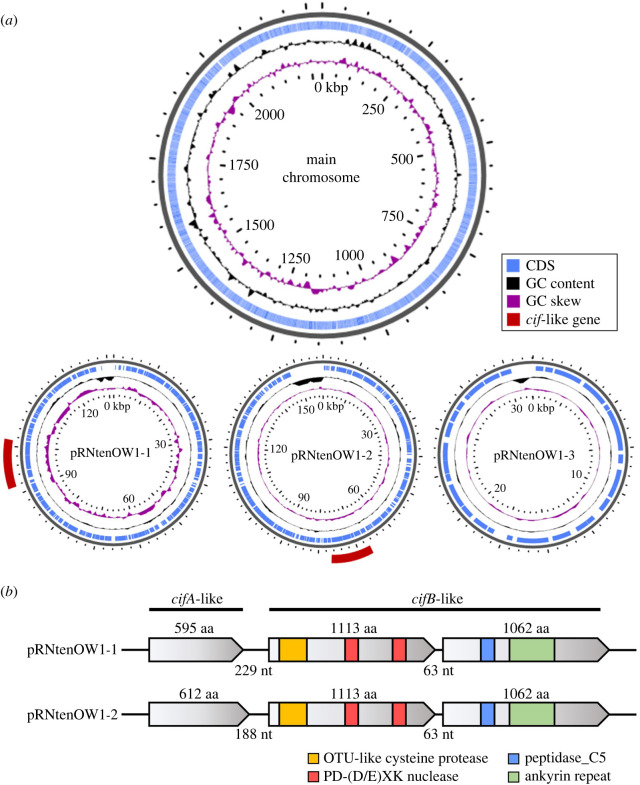
Genome structure of *Rickettsia r*Nten-OW1. (*a*) Main chromosome and three plasmids (pRNtenOW1-1 to 3) of *r*Nten-OW1. Two loci on pRNtenOW1-1 and 2 that are homologues of *cif* genes are highlighted in red. (*b*) Structures of the *cif* gene homologues found in pRNtenOW1-1 and -2. The predicted protein motifs are indicated by colour.

As reported by Shibata *et al*. [34], resequencing analysis of the OW1 line confirmed that the genome of the *Rickettsia r*Nten-OW1 strain derived from the OW1 line consisted of a main chromosome and three plasmids (designated pRNtenOW1-1 to 3, corresponding to the reference plasmids pRNTEN_1 to 3; figure 3*a*). We found *Wolbachia cif* gene homologues in pRNtenOW1-1 and -2 (figure 3*a*). The pRNtenOW1-1 encoded homologues of CifA (595 amino acids) and two CifB proteins (CifB-like-1: 1113 amino acids; CifB-like-2: 1062 amino acids; figure 3*b*). The pRNtenOW1-2 encoded a CifA homologue (612 amino acids) distinct from that in pRNtenOW1-1, but the two CifB homologues found in pRNtenOW1-2 were identical to those in pRNtenOW1-1. The plasmid-encoded CifB-like proteins were predicted to encode PD-(D/E)XK nuclease and peptidase C5 (belonging to proteases of the clan CE, which is associated with bacterial deubiquitinase; InterPro ID: IPR000855) [42], which are commonly encoded by *cif* genes in CI-inducing *Wolbachia* (figure 3*b*). In addition, CifB-like proteins contained the ovarian tumour-like (OTU-like) cysteine protease domain, which is predominantly found in eukaryotes and which primarily functions as a deubiquitinase [47], and the ankyrin repeat domain (figure 3*b*). Domain prediction analysis of the plasmids revealed the presence of transposase elements in the vicinity of the *cif* homologues (electronic supplementary material, figure S1).

### (d) Molecular phylogeny of *cif* genes

**Figure 4.**
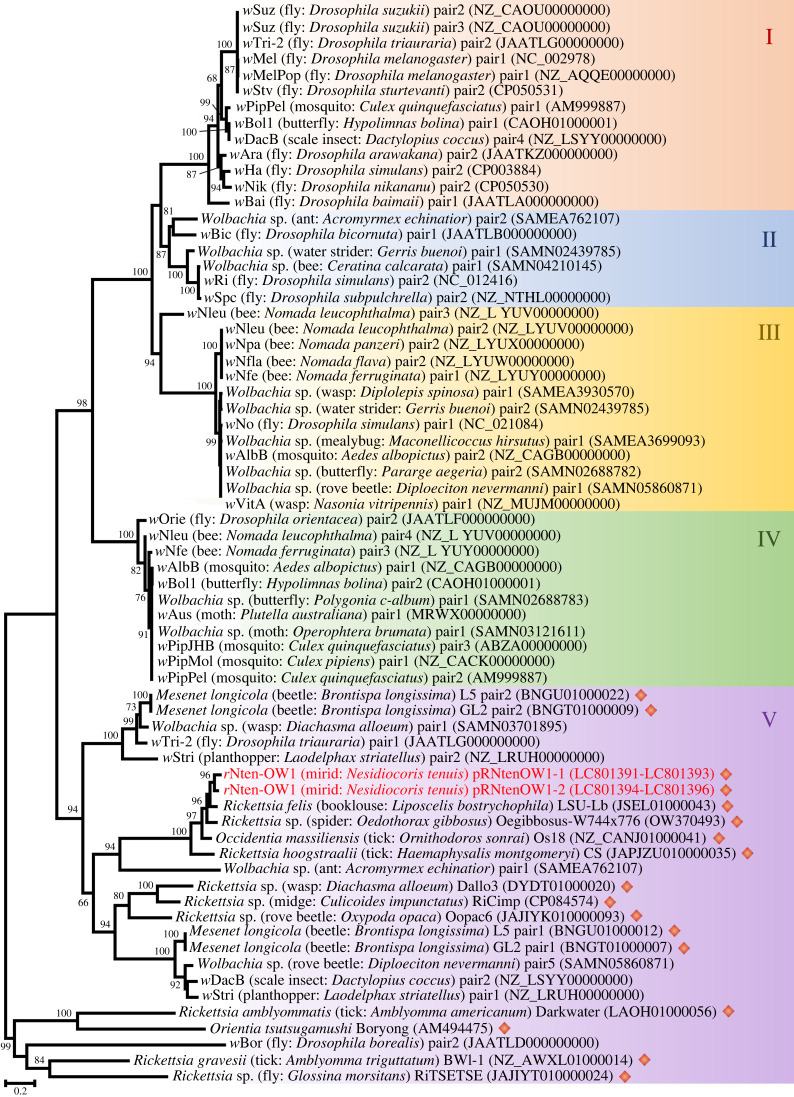
Molecular phylogeny of *cifA* and *cifB.* The tree was inferred using the maximum-likelihood method with the general time-reversible model based on 1084 positions in the aligned dataset of 68 operational taxonomic units (OTUs). The tree is midpoint-rooted. Numbers at nodes represent bootstrap values (greater than 60%) calculated from 1000 replicates. Each OTU is labelled with the strain name of the bacterium, the common and Latin names of the host insect species and the sequence accession number. Rhomboid symbols indicate *cif* homologues found in bacteria other than *Wolbachia*. Previously defined *cif* types I–V are labelled and shaded in different colours. The OTUs identified in this study are presented in red.

Concerning the phylogeny of *cif* gene sequences derived from Rickettsiales (1084 nucleotides in the final dataset), the two *cif* sequences from *r*Nten-OW1 were closely related to that from *Rickettsia felis* LSU-Lb strain (figure 4). The *cif* genes have thus far been classified into five clusters (i.e. types I–V) [20] and the *r*Nten-OW1-encoded *cif* genes were clustered into type V, consistent with a previous report that the *cif* genes possessed by Rickettsiales other than *Wolbachia* all belong the type V clade [20,43].

### (e) Comparison of *Rickettsia* and *cif* phylogenies

**Figure 5.**
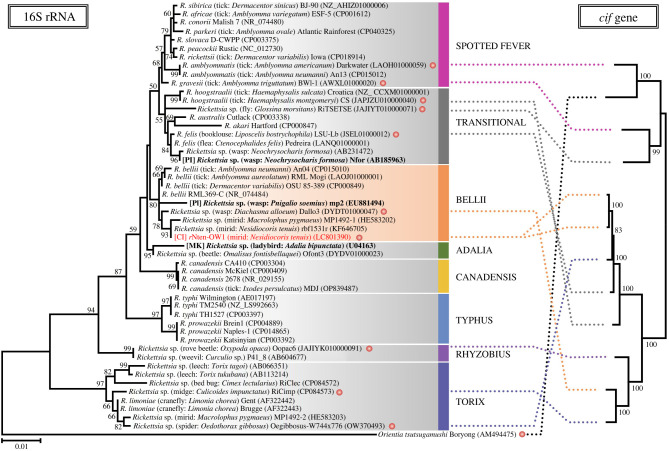
Comparison of phylogenetic trees based on the 16S rRNA and *cif* genes of *Rickettsia*. The tree on the left side was inferred by the maximum-likelihood method using the Kimura 2-parameter model based on 1336 positions in the aligned 16S rRNA sequence dataset. Each operational taxonomic unit (OTU) is labelled with the bacterial species and strain name, the common and Latin name of the host insect and the accession number of the sequence. PI, MK and CI represent *Rickettsia* isolates causing parthenogenesis induction, male-killing and cytoplasmic incompatibility, respectively. A red filled circle represents the *Rickettsia* strain with at least one homologue of the *cif* genes. Each *Rickettsia* clade is colour-labelled and the Bellii clade is shaded orange. The OTUs identified in this study are presented in red. *Orientia tsutsugamushi* was used as an outgroup. The tree on the right side was inferred from 2005 positions in the aligned dataset of *cifA* and *cifB* sequences using the maximum-likelihood method based on the general time-reversible model. The tree is midpoint-rooted. The corresponding OTUs in both trees are connected by dotted lines. Numbers at nodes represent bootstrap values (greater than 50%) calculated from 1000 replicates.

The 16S rRNA sequence (1494 bp) of *r*Nten-OW1 was identical to that of *Rickettsia* from *N. tenuis* in the Israeli population and was closely related to *Rickettsia* from the mirid bug, *Macrolophus pygmaeus*, and all of these sequences clustered in the Bellii clade of *Rickettsia* (figure 5). The phylogenetic tree of the 16S rRNA was not congruent with the tree of *cif* genes derived from diverse groups (Spotted fever, Transitional, Bellii, Rhyzobius and Torix) within the *Rickettsia* genus (figure 5).

## 4. Discussion

This study demonstrated that *Rickettsia r*Nten-OW1 induces strong CI in its native host *N. tenuis*. Specifically, no eggs hatched in crosses of untreated *N. tenuis* males (*Rickettsia*-positive) and antibiotic-treated *N. tenuis* females (*Rickettsia*-negative), both of which originated from a wild-caught female. The fact that *Rickettsia* can induce CI broadens *Rickettsia*-induced reproductive manipulations to three phenotypes: MK, PI and CI. CI is well studied as a *Wolbachia* phenotype, and the type I, II, III and IV *cif* genes possessed by *Wolbachia* have been confirmed experimentally to induce CI [15,16,48,49]. Although some *Rickettsia* species such as *R. gravesii*, *R. amblyommatis* and *R. felis* LSU-Lb carry type V *cif* homologues, their phenotypes in hosts have not been clarified [20,42]. The finding of type V *cif* homologues in the CI-inducing *Rickettsia r*Nten-OW1 genome suggests that this gene is responsible for *Rickettsia*-induced CI. To draw conclusive evidence, functional validation of *Rickettsia*-encoded *cif*-like genes in a transgenically expressing host is necessary.

It is well known that *Rickettsia* often carry plasmids. Recent studies revealed that some *Wolbachia* strains also have plasmids [20,43,50]. It is noteworthy that the *cif*-like genes of *r*Nten-OW1 were encoded in the plasmids. In addition, *cif*-like genes were also found in plasmids in other *Rickettsia* strains, such as *R. felis* LSU-Lb and RiCimp and the *Wolbachia* strain WOLB1166 [43]. *cif* genes are believed to be transferred between *Wolbachia* strains via phage integration (lysogenization) [17]. Although the evolutionary origin of *cif* genes remains unclear, the plasmids might have facilitated horizontal transfer of *cif* genes between distantly related symbionts, leading to acquisition of the CI phenotype [42]. In fact, it is common for insects to be co-infected with two or more symbionts; e.g. in Miridae species, *N. tenuis* can be co-infected with *Rickettsia*, *Wolbachia* and *Spiroplasma* [27], while *M. pygmaeus* is co-infected with *Wolbachia* and two types of *Rickettsia* [51]. These coexistence situations may provide opportunities for horizontal transmission of *cif* genes via plasmids. Alternatively, transposases present in the vicinity of the *cif* genes may allow horizontal plasmid-to-plasmid or plasmid-to-genome transfer [52,53]. Gillespie *et al*. [42] proposed that the V-type *cifB* genes could serve as a molecular platform for other types of *cifB* genes, owing to its relatively long and diverse domains. However, the origin of its acquisition remains unknown.

Although the symbionts inducing strong CI should be fixed in the host population [54–56], the frequency of *Rickettsia* infection varied widely from 21 to 96% in Japanese *N. tenuis* populations [27]. This intermediate prevalence of *Rickettsia* can cause incompatible crosses of *N. tenuis* in agricultural fields, which may result in reduced efficacy of the biological control agent. The reasons for the non-fixation of CI-inducing *Rickettsia* in *N. tenuis* are unclear, but factors other than CI, such as fitness costs or the temperature susceptibility of *Rickettsia*, could explain their limited infection frequencies in *N. tenuis*. In fact, the frequency of *Rickettsia* infection in *N. tenuis* was correlated with temperature in Japanese populations [27]. Because *N. tenuis* does not diapause under short-day conditions and does not acclimate to low temperatures [57], the population bottleneck during winter and immigration from other populations could also affect the infection frequency in these populations. While the presence/absence of *Rickettsia* does not appear to be significantly associated with the other co-infecting symbionts such as *Wolbachia* and *Spiroplasma* [27], it could be influenced by other cytoplasmic elements, such as mitochondria or viruses, or by host genotypes related to immunity or reproduction.

Similarly to other reproductive manipulating symbionts such as *Wolbachia* and *Spiroplasma*, *Rickettsia* has a wide range of host arthropods [1]. In particular, the *R. bellii* group (including *r*Netn-OW1) is one of the most common symbionts found in insects such as whiteflies, aphids, wasps, mosquitoes, beetles, planthoppers and lacewings [4,6,58–62]. The high infection frequencies of *Rickettsia* often found in these insects could be explained by the CI phenotype, in addition to their mutualistic phenotypes [63,64] and plant-mediated horizontal transmission [65].

## 5. Conclusion

For a long time, it was believed that only *Wolbachia* and *Cardinium* could induce CI. In recent years, *Rickettsiella* [12], *Mesenet* [10,11] and *Spiroplasma* [14] were demonstrated to induce CI, as observed for *Rickettsia* in the present study. Our discovery of *cif*-like genes encoded in the plasmids of CI-inducing *Rickettsia* highlights the possible occurrence of plasmid-mediated horizontal transfer of *cif*-like genes between *Wolbachia* and *Rickettsia*. We expect that such events might have allowed the acquisition of CI-inducing ability in previously non-CI-inducing symbionts.

## Data Availability

The sequence read data were deposited in the DDBJ under the accession numbers PRJDB17463, PRJDB17616 and LC801390–LC801396. Any additional information required to reanalyse the data reported in this paper is accessible from the Dryad Digital Repository: https://doi.org/10.5061/dryad.t1g1jwt9b [66]. The data are provided in electronic supplementary material [67].
